# Medienberichterstattung zu Präventions- und Therapiemöglichkeiten an den Beispielen Diabetes mellitus und Depression

**DOI:** 10.1007/s00103-020-03250-4

**Published:** 2020-11-30

**Authors:** Doreen Reifegerste, Annemarie Wiedicke, Linn Julia Temmann

**Affiliations:** Philosophische Fakultät, Seminar für Medien- und Kommunikationswissenschaft, Universität Erfurt, Nordhäuser Str. 63, 99089 Erfurt, Deutschland

**Keywords:** Medienberichterstattung, Diabetes mellitus, Depression, Prävention, Therapie, Media coverage, Diabetes mellitus, Depression, Prevention, Therapy

## Abstract

**Hintergrund:**

Diabetes mellitus und Depression sind Erkrankungen mit einer steigenden Prävalenz in Deutschland. Die Bevölkerung über die entsprechenden Präventions- und Therapiemöglichkeiten zu informieren ist beispielsweise durch journalistische Medienberichterstattung möglich. Denn Untersuchungen zeigen, dass mediale Darstellungen von Präventions- und Behandlungsmöglichkeiten das Gesundheitsverhalten, aber auch die Zustimmung zu bestimmten Maßnahmen und somit die strukturelle Gesundheitsversorgung beeinflussen können.

**Ziel der Arbeit (Fragestellung):**

Über die Berichterstattung deutscher Medien zur Vorbeugung und Behandlung von Diabetes mellitus und Depressionen ist bislang nur wenig bekannt. Diese Arbeit untersucht, wie diese beiden Erkrankungen in deutschen Medien dargestellt werden. Der Fokus liegt hierbei auf den Präventions- und Therapieoptionen.

**Material und Methoden:**

Es wurde eine quantitative Inhaltsanalyse von deutschen Qualitätsmedien (Print und Online) im Zeitraum 2012–2018 durchgeführt. Insgesamt wurden *N* = 645 Artikel analysiert, *n* = 219 davon zum Thema Diabetes mellitus und *n* = 426 zum Thema Depression.

**Ergebnisse und Diskussion:**

Diabetes mellitus und Depression sind trotz ihrer hohen Prävalenz nur selten Hauptthema in der deutschen Medienberichterstattung. Über Depression wird deutlich häufiger berichtet als über Diabetes mellitus – oftmals jedoch im Zusammenhang mit Suizid oder Prominenten. Bei Diabetes mellitus differenzieren Berichte nur unzureichend zwischen den unterschiedlichen Typen. Eine medikamentöse Therapie wird bei beiden Erkrankungen häufiger erwähnt als niedrigschwellige Maßnahmen und (strukturelle) Präventionsoptionen, was sich ungünstig für Hilfesuchende auswirken kann. Um Prävention und niedrigschwellige Behandlungsformen als Lösungen in der öffentlichen Wahrnehmung zu etablieren, sollten strategische Kommunikator*innen diese stärker in ihrer Pressearbeit fokussieren.

## Abstract (en)

**Background:**

Diabetes mellitus and depression are diseases with a rising prevalence in Germany. Journalistic media coverage offers the opportunity to inform the public about options for prevention and therapy. Research shows that media portrayals of different prevention and treatment options might influence health behavior as well as policy support and eventually structural healthcare.

**Objectives:**

To date, little is known about the media coverage of the prevention and treatment of diabetes mellitus and depression. This study aims at demonstrating how diabetes mellitus and depression are portrayed in the German news media, focusing on options for prevention and therapy.

**Materials and methods:**

A quantitative content analysis of German print and online media from 2012 to 2018 was conducted. In sum, *N* = 645 articles on diabetes mellitus (*n* = 219) and depression (*n* = 426) were analyzed.

**Results and discussion:**

Diabetes mellitus and depression are rarely the main subject in German news coverage despite their high prevalence. Depression is reported more frequently than diabetes mellitus – often, however, in the context of suicide or celebrities. Regarding diabetes mellitus, reports differentiated insufficiently between the various types. For both conditions, pharmacotherapy was mentioned more frequently than low-threshold measures and structural prevention opportunities, which might have adverse consequences for those seeking help. Overall, to establish prevention and low-threshold forms of therapy as treatment options in the public, strategic communicators should focus more on them.

## Hintergrund

### Darstellung von Krankheiten in den Medien

Die verschiedenen Beiträge dieser Ausgabe des Bundesgesundheitsblattes zeigen, dass die Berichterstattung in den Medien die Einstellungen, Verhaltensweisen und den Gesundheitszustand der Gesamtbevölkerung beeinflussen kann. Dies ist vor allem relevant für das Wissen, die Akzeptanz und die Umsetzung von Präventions- und Behandlungsprogrammen [1], da sich die Bevölkerung vor allem in den Medien über die Prävention, Symptome und die Behandlung von Krankheiten informiert [2]. Gleichzeitig beeinflussen mediale Darstellungen von Krankheiten die öffentliche Meinung sowie die Gesundheitskompetenz [3–6]. Daher spielt es eine wichtige Rolle, worauf Medien in ihrer Berichterstattung fokussieren (siehe dazu auch den Beitrag von Daube und Ruhrmann in diesem Themenheft). Je nachdem, welche Präventionsmöglichkeiten oder Therapien Medienberichte in den Blick nehmen, kann dies auch den Umgang mit einer Krankheit bei politischen Entscheidungsträger*innen, Kostenträgern, den Betroffenen oder ihren Angehörigen verändern. Wirkungsstudien zu diesen unterschiedlichen Schwerpunktsetzungen (d. h. Frames) in der Berichterstattung über Depression haben beispielsweise gezeigt, dass die daraus resultierende Wahrnehmung über die Präventions- und Behandlungsmöglichkeiten auch die Unterstützung von öffentlichen Gesundheitsprogrammen und das zivile Engagement beeinflusst [5, 7].

Darüber hinaus haben mediale Darstellungen auch Einfluss auf das Selbstmanagement von Betroffenen sowie den Umgang mit Betroffenen durch andere Personen. So können Mediendarstellungen von Depressionen die Betroffenen und ihre Behandlungsbereitschaft sowie den Therapieerfolg negativ beeinflussen [1]. Anhand dieser verschiedenen Wirkungen, die von der medialen Berichterstattung zu Krankheiten ausgehen können, zeigt sich die bedeutende Rolle der Medienberichterstattung für die Informationsverbreitung und Meinungsbildung über den Umgang mit diesen Krankheiten [8].

Die Analyse von Gesundheitsthemen in der Berichterstattung deckt wiederholt auf, dass Mediendarstellungen nur bedingt mit der Realität (z. B. der epidemiologischen Gesundheitsberichterstattung) oder medizinischen Empfehlungen (z. B. den Leitlinien) übereinstimmen und damit den Qualitätsansprüchen eines evidenzbasierten Medizinjournalismus bzw. evidenzbasierter Gesundheitsinformationen nicht gerecht werden [9, 10]. Verzerrte Medieninhalte können problematische Effekte haben, da ihre Rezeption auch zu einer verzerrten Wahrnehmung der Realität führen kann [11]. Dabei wird immer wieder deutlich, dass schwerwiegende Krankheiten, die mit hoher Mortalität und dramatischen Krankheitsverläufen (wie das etwa bei Krebserkrankungen häufig der Fall ist) verbunden sind, sehr viel präsenter in den Medien auftreten als alltägliche Gesundheitsprobleme mit weniger schwerwiegenden Symptomen.

Dies gilt gleichermaßen für die Erkrankung von prominenten Personen, die zu einer deutlichen Zunahme der Berichterstattung über ein Gesundheitsthema führt, ohne dass sich an der Prävalenz etwas geändert hätte. Dies liegt vor allem daran, dass Journalist*innen sich bei der Auswahl von Nachrichteninhalten weniger an der Gesundheitsberichterstattung, sondern vielmehr an den Interessen ihres Publikums bzw. ihrer Arbeitgeber (d. h. den Herausgebenden oder Verlagen) orientieren [12]. Kommunikationswissenschaftliche Untersuchungen unterstreichen, dass Journalist*innen bestimmte Zielstellungen verfolgen, wenn sie über Gesundheitsthemen berichten. Diese Zielstellungen begründen sich vor allem aus ihrem Rollenverständnis. Gesundheitsjournalist*innen verstehen sich dabei z. B. als Multiplikatoren, als Interpretierende, als Skeptiker*innen oder als Lösungsgebende, was wiederum zu unterschiedlichen Schwerpunkten und damit ggf. auch unterschiedlichen Verzerrungen in ihrer Berichterstattung führen kann [13]. Lösungsgeber*innen setzen sich bspw. am ehesten aktiv für die Themen, über die sie berichten, ein, indem sie Lösungen anbieten oder auch den Diskurs zu einem bestimmten Thema anregen wollen [13]. Dieses Rollenverständnis orientiert sich am ehesten an Vorstellungen des sog. konstruktiven Journalismus, der nicht nur Probleme und Missstände darstellen, sondern vielmehr den Blick auf Lösungsansätze und Handlungsmöglichkeiten lenken will. Entsprechende Wirkungsstudien legen nahe, dass diese Art der Berichterstattung zu mehr Zuversicht und höherer Bereitschaft zum Austausch über Lösungen sowie gesellschaftlichem Engagement beiträgt [14].

Darüber hinaus hängen ihre Zielstellungen auch mit den (ökonomischen) Rahmenbedingungen der Redaktionen bzw. der Medieninstitutionen zusammen. Dadurch können Nachrichtenfaktoren wie Konflikt, Negativität oder Prominenz einen wichtigen Einfluss auf die Auswahl und Darstellung von Medieninhalten nehmen, da sie (im Gegensatz zur neutralen oder realitätsnäheren Darstellung) das (Kauf‑)Interesse des Publikums steigern [12]. Zudem sind manche Gesundheitsthemen – etwa bestimmte psychische Störungen – mit Stigmata (d. h. sozialer Ächtung) verbunden, sodass sie generell seltener oder aber in stigmatisierenden Kontexten dargestellt werden – wie beispielsweise im Zusammenhang mit Kriminalität und Gewalt [15, 16].

Vor diesem Hintergrund analysiert der vorliegende Beitrag die mediale Berichterstattung zu Diabetes mellitus und Depression als Gesundheitsthemen mit hohen Prävalenzzahlen.

### Diabetes mellitus und Depression als Medienthema

Erkrankungen mit schleichenden Verläufen und weniger aufsehenerregenden Symptomen scheinen aufgrund ihres geringen Nachrichtenwertes eher wenig im Fokus der Medienberichterstattung zu stehen. Empirische Daten, die diese Annahme untermauern würden, liegen für die deutsche Medienberichterstattung bislang kaum vor. Fraglich ist daher, welchen Stellenwert derartige Krankheiten in der Berichterstattung in Deutschland erhalten und mit welchem Fokus sie dargestellt werden. Dies soll exemplarisch an den Gesundheitsthemen Diabetes mellitus und Depression untersucht werden, zwei Erkrankungen mit hoher und steigender Prävalenz, die jeweils auch mit hohen Gesundheitsausgaben verbunden sind [17, 18]. Aufgrund des medizinischen Fortschritts und der zunehmend besseren Versorgung der chronisch Erkrankten werden deren Lebenserwartung und somit die Prävalenzzahlen der jeweiligen Erkrankung wahrscheinlich weiter steigen. Folglich werden auch die Versorgungsbedarfe betroffener Menschen weiter zunehmen [19]. Darüber hinaus ist bei beiden Erkrankungen von einer deutlichen Anzahl an Personen ohne Diagnose auszugehen [20, 21].

Diabetes mellitus ist eine weitverbreitete chronische Stoffwechselkrankheit, bei der hauptsächlich zwischen Typ-1-Diabetes (tritt bereits im Kindes- oder Jugendalter auf) und Typ-2-Diabetes (meist erst im mittleren bis höheren Erwachsenenalter diagnostiziert) unterschieden wird [22]. Diabetes mellitus Typ 2 erreicht in der Altersgruppe der über 65-Jährigen eine 12-Monats-Prävalenz von 17,6 % unter Frauen und 21,1 % bei Männern [17]. Insgesamt leben in Deutschland etwa 6,9 Mio. Menschen, die von Diabetes mellitus Typ 2 betroffen sind, sowie 372.000 Typ-1-Diabetiker*innen [23]. Für Prävention und Behandlung insbesondere von Diabetes Typ 2 haben sogenannte Lebensstilfaktoren wie Bewegung und Ernährung eine wichtige Bedeutung [24, 25].

Depressive Störungen gehören weltweit zu den häufigsten psychischen Erkrankungen [26] und treten in unterschiedlichen Lebensphasen (z. B. Altersdepression, postnatale Depression) und Zusammenhängen (z. B. Arbeitsplatz, posttraumatische Belastung) auf. In Deutschland erkrankt etwa ein Fünftel einmal im Leben daran und etwa 5,3 Mio. Erwachsene sind damit diagnostiziert [27]. Etwa 6 % berichteten von einer ärztlich oder psychotherapeutisch diagnostizierten Depression (12-Monats-Diagnosen), wobei nicht alle Formen chronisch verlaufen [28]. Neben Medikamenten und Psychotherapie haben auch Bewegung, Ernährung, Entspannung, Psychoedukation sowie die soziale Unterstützung durch Angehörige Einfluss auf den Behandlungserfolg von Depressionen in verschiedenen Altersgruppen [29–32].

Dementsprechend zielen zahlreiche Regierungsprogramme und Maßnahmen von Krankenversicherungen (z. B. das nationale Gesundheitsziel „Depressive Erkrankungen“ oder die Disease-Management-Programme für chronische Erkrankungen) darauf ab, diesen Erkrankungen durch die Förderung eines gesunden Lebensstils vorzubeugen bzw. sie nachhaltig zu behandeln [20]. Letztlich muss dabei berücksichtigt werden, dass die individuellen Maßnahmen häufig nur von den Betroffenen angewendet werden können, wenn die strukturellen Bedingungen hierfür vorhanden sind. Das heißt beispielsweise, dass gesunde Ernährung und ausreichend körperliche Aktivität nur dann möglich sind, wenn gesunde Lebensmittel für die Betroffenen auch bezahlbar und erkennbar sind oder wenn der Zugang zu Sporteinrichtungen oder öffentlichen Plätzen dafür auch gegeben ist.

### Forschungsstand zur Berichterstattung über Diabetes mellitus und Depression

Bisherige Inhaltsanalysen zu Diabetes mellitus und Depression stammen mehrheitlich nicht aus Deutschland und sind (aufgrund unterschiedlicher Gesundheitssysteme und Gesundheitskonzepte) daher nur bedingt geeignet, um Aussagen über die deutsche Berichterstattung abzuleiten.

Aus Inhaltsanalysen von Artikeln der *New York Times* [8] und von Printmedien in Neuseeland [33] wird deutlich, dass ein großer Teil der Berichterstattung nicht zwischen den verschiedenen Arten des Diabetes mellitus unterscheidet, was zu Verwirrungen in Bezug auf die Präventions- und Therapiemöglichkeiten führen kann. Gollust und Lantz [34] zeigen in einer Inhaltsanalyse der US-amerikanischen Berichterstattung, dass die Beiträge mehrheitlich Übergewicht, ungesunde Ernährung und mangelnde Bewegung als Ursachen der Erkrankung nennen. Vielfach werden diese Risikofaktoren auch mit Blick auf eine Verhaltensänderung als Präventions- und Therapiemöglichkeit genannt. Allerdings stellt nur eine Minderheit der Beiträge dann auch entsprechende Programme zur Gesundheitsförderung und zum Gesundheitsmanagement vor, während gleichzeitig Medikamente in mehr als der Hälfte der Beiträge als Therapieoption angeführt werden.

Auch die Inhaltsanalysen zur Darstellung von Depressionen in US-amerikanischen und chinesischen Medien [3, 35] machen deutlich, dass die Medienberichterstattung überwiegend medizinische Behandlungsoptionen (wie Medikamente und Psychotherapie) aufzeigt. Hingegen werden Möglichkeiten der Prävention und gesellschaftlich-politische Einflussfaktoren vernachlässigt. Wie auch für Diabetes mellitus zeigt sich eine begriffliche Undifferenziertheit. Aus einer qualitativen Inhaltsanalyse überregionaler Tageszeitungen in Deutschland geht hervor, dass häufig das Phänomen Burn-out mit den Symptomen einer Depression beschrieben wird [36]. Die Autor*innen der Studie vermuten, dass Burn-out durch den Arbeitskontext und die zahlreichen prominenten Betroffenen nahbarer ist als eine Depression, was Vor- und Nachteile hinsichtlich der Stigmatisierung (d. h. der sozialen Anerkennung) dieser Krankheit haben kann. Eine Analyse der deutschen Berichterstattung zu Depression von 2003 resümierte zudem, dass die untersuchten Artikel mehrheitlich (aus medizinischer Sicht) sehr unzureichend über die Krankheit berichteten [37].

## Ziel der Arbeit (Fragestellung)

Bislang ist somit weitgehend unklar, welchen Stellenwert Diabetes mellitus und Depressionen in der Berichterstattung in Deutschland einnehmen und welche Aspekte dabei fokussiert werden. Der vorliegende Beitrag zielt daher mit seinen Forschungsfragen (FF) darauf ab, zu untersuchen, inwieweit die Gesundheitsthemen Diabetes mellitus und Depression in der deutschen Berichterstattung präsent sind (FF1), welche Präventionsmöglichkeiten (FF2) und welche Therapieoptionen (FF3) aufgezeigt werden. Daraus lassen sich wichtige Implikationen für die strategische Kommunikation von Gesundheitsbehörden und Präventionsinstitutionen ableiten.

## Material und Methoden

Um die Forschungsfragen zu beantworten, wurde eine quantitative Inhaltsanalyse der Berichterstattung über Depressionen und Diabetes mellitus in überregionalen Printmedien und deren entsprechenden Onlineangeboten in Deutschland durchgeführt. Um auf jene Leitmedien zu fokussieren, die vermutlich die öffentliche Meinungsbildung durch ihren Einfluss auf die Entscheidungsträger*innen am deutlichsten prägen [38], wurden für die Analyse Artikel im Zeitraum 2012 bis 2018 aus den wichtigsten deutschen überregionalen Qualitätszeitungen des gesamten politischen Spektrums ([39]; die Welt, FAZ, SZ, taz) sowie die Medienberichterstattung im Nachrichtenmagazin bzw. der Wochenzeitung mit den höchsten Auflagenzahlen ([40, 41]; Spiegel und Zeit) untersucht.

Um die Artikel zu identifizieren, wurde eine Stichwortsuche (*depress*, *diabet*) in verschiedenen Datenbanken (LexisNexis, FAZ Archiv, SZ Archiv) durchgeführt. Es wurden nur redaktionelle Beiträge eingeschlossen, die sich im Hauptthema^1^ mit Diabetes mellitus bzw. Depressionen auseinandersetzen, was final zu *N* = 645 Artikeln führte, wovon *n* = 244 aus den Printversionen und *n* = 401 aus den Onlineversionen der Medien stammen. Wenn Depression als Komorbidität von Diabetes mellitus (nur in Einzelfällen) genannt wurde, wurden solche Artikel dem Hauptthema Diabetes mellitus zugeordnet.

Das Reliabilitätsmaß Krippendorffs Alpha als Kenngröße zur Güte der Messung (Vergleich der Messungen der einzelnen untersuchten Variablen zwischen verschiedenen Codierer*innen, um die Replizierbarkeit der Ergebnisse sicherzustellen [42]) ist mit einem Wert von ≥0,77 ausreichend hoch. Neben dem Hauptthema (Depression, Burn-out; Diabetes mellitus allgemein, Typ-1-Diabetes, Typ-2-Diabetes, Schwangerschaftsdiabetes) wurden jeweils die Präventionsmaßnahme (allgemein; Bewegung, Sport bzw. körperliche Aktivität; gesunde Ernährung; Achtsamkeit, Work-Life-Balance, staatliche bzw. institutionelle Bewegungs- und Ernährungsprogramme; Krippendorff’s Alpha ≥0,79) und die Therapiemöglichkeit (allgemein; Bewegung, Sport bzw. körperliche Aktivität; Abnehmen, Gewichtsreduzierung; Ernährung, Ernährungsumstellung; Psychotherapie; Klinikaufenthalte; Selbsthilfegruppen on- und offline; technische Hilfsmittel; Krippendorff’s Alpha ≥0,87) erhoben.

## Ergebnisse

### Präsenz der Themen (Forschungsfrage 1)

Im Überblick der Berichterstattung zeigt sich, dass die Anzahl der Artikel über depressive Erkrankungen (*n* = 380; 58,9 %) und Burn-out (*n* = 46; 7,1 %) deutlich höher liegt als die Anzahl der Beiträge über Diabetes mellitus (*n* = 219; 34,0 %). Dabei zeigt sich, dass die Beiträge vielfach (*n* = 94; 14,6 %) mehrere Arten von Diabetes mellitus darstellen oder die verschiedenen Arten nicht unterscheiden, während nur ganz selten explizit zu Typ-1-Diabetes (*n* = 50; 7,8 %), Typ-2-Diabetes (*n* = 70; 10,9 %) oder Schwangerschaftsdiabetes (*n* = 5; 0,8 %) berichtet wird.

**Figure 1:**
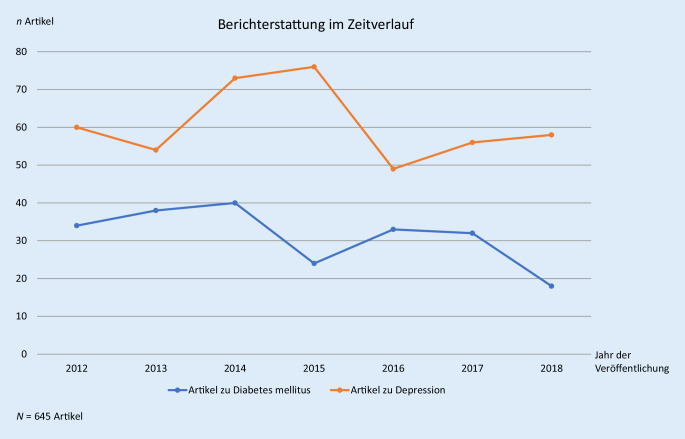

Im Zeitverlauf von 2012 bis 2018 wird deutlich, dass es jedes Jahr deutlich mehr Beiträge zu Depressionen als Diabetes mellitus gibt, wobei Letztere tendenziell abnehmen (Abb. 1).

Bei einer näheren Betrachtung der Artikel wird deutlich, dass diese häufig (*n* = 41; 6,4 %) in Zusammenhang mit der Erkrankung von Prominenten stehen. Insgesamt treten in 115 aller 645 Artikel (17,8 %) prominente Betroffene auf, vor allem in der Depressionsberichterstattung (*n* = 103). Neben Robert Enke (*n* = 39; 6,0 % der Gesamtberichterstattung) wird dabei auch über weitere Personen aus dem Leistungssport (in insgesamt *n* = 71 Beiträgen; 11,0 %) berichtet, darunter u. a. Andreas Biermann, Lindsay Vonn und Michael Phelps.

### Präventionsmaßnahmen (Forschungsfrage 2)

Präventionsmaßnahmen zur Vorbeugung von Diabetes mellitus und Depression nehmen in der medialen Berichterstattung eher eine untergeordnete Rolle ein (Tab. 1). Insgesamt thematisiert nur ein knappes Fünftel aller Beiträge Präventionsmöglichkeiten (*n* = 140; 21,7 %). Krankheitsspezifisch zeigt sich jedoch, dass bei den Diabeteserkrankungen – bis auf eine Ausnahme – anteilig die Präventionsmaßnahmen deutlich häufiger genannt werden als in Beiträgen zum Thema Depression. Die am häufigsten dargestellten Präventionsmaßnahmen bei Diabetes mellitus sind Bewegung und gesunde Ernährung, während dies bei Depression Achtsamkeit und eine gesunde Work-Life-Balance sind. Diese Unterschiede sind laut Chi-Quadrat-Test signifikant.

**Table 1:** 

	Diabetes mellitus *(n* = 219)		Depression *(n* = 426)		Gesamt (*N* = 645)		Chi-Quadrat	
	*N*	%	*n*	%	*n*	%	*φ*	*p*
Prävention wird thematisiert	68	31,1	72	16,9	140	21,7	0,163	$<$0,001
Präventionsmaßnahmen im Detail								
Bewegung, Sport bzw. körperliche Aktivität	34	15,5	11	2,6	45	7,0	0,247	$<$0,001
Gesunde Ernährung	34	15,5	4	0,9	38	6,0	0,299	$<$0,001
Achtsamkeit, Work-Life-Balance	1	0,5	21	4,9	22	3,4	0,117	0,003
Staatliche bzw. institutionelle Bewegungs- und Ernährungsprogramme	10	4,6	3	0,7	13	2,0	0,132	0,001
Offene Nennung: Präventionsgesetz	0	0	2	0,5	2	0,3	–	–

*Anmerkung*. Mehrfachnennungen der Präventionsmaßnahmen möglich

### Therapiemöglichkeiten (Forschungsfrage 3)

Behandlungsoptionen werden bei beiden Erkrankungen in der Mehrheit der Artikel thematisiert und unterscheiden sich je nach Erkrankung (Tab. 2). Zu den meistgenannten Therapiemaßnahmen in der Berichterstattung bei Depressionen zählen Psychotherapien, die Einnahme von Medikamenten und Klinikaufenthalte. Als Behandlungsmöglichkeiten bei Diabetes mellitus nennt die Berichterstattung am häufigsten die Einnahme von Medikamenten und die Ernährung bzw. eine Ernährungsumstellung. Bei beiden Erkrankungen werden Bewegung, Sport und körperliche Aktivität als weitere Lösungen dargestellt, um die Krankheit erfolgreich zu managen.

**Table 2:** 

	Depressionen (*n* = 426)		Diabetes (*n* = 219)		Gesamt (*N* = 645)		Chi-Quadrat	
	*N*	%	*n*	%	*n*	%	*φ*	*p*
Therapiemöglichkeiten werden thematisiert	279	65,5	148	67,6	427	66,2	0,021	0,596
Therapieoptionen im Detail								
Einnahme von Medikamenten	156	36,6	126	57,5	353	54,7	0,195	$<$0,001
Psychotherapie	176	41,3	5	2,3	181	28,1	0,427	$<$0,001
Bewegung, Sport, körperliche Aktivität	40	9,4	42	19,2	82	12,7	0,135	0,001
Klinikaufenthalt	74	17,4	5	2,3	79	12,3	0,224	$<$0,001
Ernährung(sumstellung)	7	1,6	52	23,7	59	9,2	0,365	$<$0,001
Technische Hilfsmittel, z. B. Apps oder Insulinpumpen	23	5,4	36	16,4	59	9,2	0,180	$<$0,001
Selbsthilfegruppen	25	5,9	5	2,3	30	4,7	0,084	0,035
Abnehmen, Gewichtsreduzierung	0	0	26	11,9	26	4,0	0,286	$<$0,001

*Anmerkung*. Mehrfachnennungen der Therapiemöglichkeiten möglich

## Diskussion

Zunächst zeigt sich in einer Gesamtbetrachtung, dass die beiden in Deutschland hoch prävalenten Erkrankungen Diabetes mellitus und Depression nur selten als Hauptthema in der Medienberichterstattung präsent sind. Dies zeigt sich bspw. im Vergleich zur Gesamtanzahl der Beiträge, in denen Diabetes mellitus oder Depression im Betrachtungszeitraum eher beiläufig genannt werden. Im Untersuchungszeitraum finden sich dabei Beiträge über depressive Erkrankungen deutlich häufiger in der Berichterstattung als Beiträge zu Diabeteserkrankungen, obwohl in Deutschland die Prävalenz für Diabetes (rund 7 Mio. Diabetiker*innen [18]) höher ist als für Depressionen (5,3 Mio. Erwachsene mit diagnostizierten Depressionen [27]). Für diese Abweichung der Medienrealität von der epidemiologischen Gesundheitsberichterstattung lassen sich verschiedene Erklärungen anführen. Zum einen könnten dafür Entwicklungen im Gesundheitssystem verantwortlich sein. Obwohl die Prävalenz bei beiden Krankheiten ansteigt [18], weisen depressive Erkrankungen in den vergangenen Jahren einen deutlich steileren Anstieg auf, der möglicherweise auch den Nachrichtenwert Negativismus erhöht, weil es sich dabei um eine Entwicklung handelt, die besonders negativ bewertet wird und daher umso wahrscheinlicher berichtet wird.

Eine grundsätzliche Tabuisierung (d. h. grundsätzliche Nichtbeachtung) des Themas Depressionen durch die Qualitätsmedien liegt somit nicht vor. Dennoch legen die häufig individuell zentrierte Berichterstattung und der Fokus auf das Leistungsprinzip (was u. a. durch die häufige Darstellung von Leistungssportlern zum Ausdruck kommt) im Zusammenhang mit Burn-out auch eine gewisse Stigmatisierung der Erkrankung nahe. Somit lässt sich vermuten, dass Burn-out mit Stärke und Erfolg verknüpft ist und – im Vergleich zu Depressionen – gesellschaftlich stärker anerkannt wird [36].

Zum anderen könnten die Ursachen für die höhere Präsenz der depressiven Erkrankungen auch in den Zielvorstellungen (d. h. dem Rollenverständnis) und den Auswahlkriterien (d. h. dem wahrgenommenen Nachrichtenwert) der Journalist*innen liegen. In Bezug auf die Zielvorstellungen der Journalist*innen lässt sich vermuten, dass weder die Orientierung an epidemiologischen Grundlagen noch die Präventionssicht prägend für die Berichterstattung sind. Vielmehr scheinen die Publikumsinteressen im Vordergrund zu stehen, denn sowohl die Betroffenheit prominenter Personen als auch die tödlichen Folgen z. B. in Form von Suizid stehen im Zusammenhang mit den Nachrichtenwerten Prominenz und Negativität. Das bedeutet, dass die Nachrichten, die im Zusammenhang mit einer berühmten Persönlichkeit stehen oder bei denen es sich um besonders schlechte Informationen handelt, mit höherer Wahrscheinlichkeit für die Medienberichterstattung ausgewählt werden [43]. Für fundiertere Aussagen über das Rollenverständnis der Journalist*innen und ihre Ziele wären allerdings Untersuchungen zu den Medienschaffenden und ihres Selektionsprozesses notwendig.

Wie in der medialen Berichterstattung über Diabetes mellitus in den Vereinigten Staaten [8] und Neuseeland [33] zeigt sich auch in Deutschland, dass nur in Einzelfällen zwischen den verschiedenen Erkrankungsformen unterschieden wird. Dies erscheint angesichts der deutlichen Unterschiede der Präventions- und Behandlungsmöglichkeiten von Typ 1 und Typ 2 unzureichend, wenn man von dem Anspruch einer realitätsnahen bzw. evidenzbasierten Medienberichterstattung ausgeht [9]. Wenn eine Unterscheidung zwischen Typ 1 und Typ 2 stattfindet, dann entspricht das Verhältnis der Berichterstattung nicht den tatsächlichen Verbreitungszahlen, da Typ 2 um ein Vielfaches prävalenter ist, während er in der Berichterstattung nur unwesentlich häufiger auftritt als Typ 1.

Präventionsmöglichkeiten oder Früherkennungsangebote (z. B. für Diabetes mellitus Typ 2 [44]) werden insgesamt nur sehr selten in der untersuchten Berichterstattung thematisiert. Ebenso werden auch umfassendere strukturelle Programme, wie bspw. die *Disease-Management*-Programme der gesetzlichen Krankenkassen zur Unterstützung des Selbstmanagements bei chronisch Erkrankten, fast gar nicht erwähnt, wenn über die Behandlungsmöglichkeiten der Krankheiten berichtet wird. Ähnliches gilt für Gesundheitskampagnen von Behörden, wie der Bundeszentrale für gesundheitliche Aufklärung (BZgA), oder auch Angebote im Rahmen der betrieblichen Gesundheitsförderung, obwohl sie Bestandteil der Prävention von Diabetes mellitus und Depressionen sind [20].

Der Mangel an der Thematisierung von Präventionsmöglichkeiten in meinungsbildenden Leitmedien ist in Hinblick auf die zunehmende und hohe Prävalenz von Diabetes mellitus und Depression, die jeweils mit langfristigen und hohen Kosten für das Gesundheitssystem verbunden sind [19], kritisch zu bewerten, wenn das Ideal eine evidenzbasierte und lösungsorientierte Berichterstattung ist [12]. Da es sich hier um Herausforderungen für das Gesundheitswesen und die gesamte Gesellschaft handelt, hätten gerade die Qualitätsmedien als bedeutsame Instanzen für die Bildung der öffentlichen Meinung [38] die Möglichkeit, die (strukturellen) Präventionsmöglichkeiten deutlich stärker aufzugreifen, wenn sie sich in ihrem Rollenverständnis (im Sinne des konstruktiven Journalismus) eher als lösungsorientierte Berater*innen, denn als Problemfokussierende verstehen [13, 20].

Bei den Therapiemöglichkeiten stehen insgesamt die medizinischen und damit auch kostenintensiven Behandlungsformen im Vordergrund. In der Medienberichterstattung über Diabetes und Depression zeigt sich eine Medikalisierung, indem überwiegend pharmakologische und gerätetechnische Therapien thematisiert werden [45]. Für die an einer Depression erkrankten Personen ist dies auch deshalb problematisch, da diese Therapieoptionen oft kurzfristig nicht verfügbar sind. Die kostengünstigeren und niedrigschwelligen Behandlungsmöglichkeiten, wie soziale Unterstützung oder körperliche Aktivität erhalten deutlich weniger Aufmerksamkeit in der Medienberichterstattung, obwohl „körperliches Training [...] bei Depressionen in einem ähnlichen Maße wirksam sein [kann] wie eine medikamentöse Therapie“ [47, S. 55] und auch für die soziale Unterstützung positive Präventionseffekte in systematischen Reviews aufgezeigt werden konnten [46]. In ähnlicher Weise werden auch digitale Angebote wie Onlineselbsthilfegruppen oder Apps zum Selbstmanagement weder als Präventions- noch als Therapiemöglichkeit häufig erwähnt, wenngleich in den letzten Jahren deutlich geworden ist, dass sie für beide Bereiche effektive und kostengünstige Bestandteile des Gesundheitssystems sein können [48].

Bei der vorliegenden Analyse muss einschränkend beachtet werden, dass lediglich die Berichterstattung der Leitmedien und deren Onlineversionen untersucht wurde. Um die Medienangebote für verschiedene Bevölkerungsgruppen (und insbesondere auch jene, die verstärkt von Risikogruppen genutzt werden) in ihrer Gänze abzubilden, sollte darüber hinaus auch die Darstellung von Krankheiten in anderen Medien, d. h. auf Webseiten, in sozialen Medien [49], im Fernsehen (Ratgebersendungen), Apps, Gesundheitsmagazinen, Büchern, aber auch Darstellungen in Unterhaltungsangeboten (bspw. in Arztserien) berücksichtigt werden. Onlineangebote bieten oft sehr viel detailliertere Informationen über Therapiemöglichkeiten und Unterstützungsangebote als Nachrichtenmedien. Allerdings werden diese Medien überwiegend von den Betroffenen genutzt, die aktiv nach diesen Inhalten suchen, während die Nachrichtenmedien auch von Personen genutzt werden, die von dem spezifischen Gesundheitsthema (vordergründig) nicht betroffen sind [2]. Zudem müssen die Medieninhalte für ein Gesamtverständnis der beeinflussenden Inhalte im Zusammenhang mit Gesundheitsinformationen aus anderen Quellen betrachtet werden. Denn neben Informationen aus den Medien sind sowohl medizinisches Personal als auch Angehörige oder andere Betroffene wichtige Quellen für Gesundheitsinformationen sowie Unterstützende bei der Einordnung von Medieninformationen [50].

Aus der vorliegenden Arbeit lässt sich jedoch für die strategische Kommunikation von Bundesbehörden oder Präventionsinstitutionen (z. B. in Form von Pressemitteilungen oder Eigenveröffentlichungen) schlussfolgern, dass Präventionsmöglichkeiten sowie kostengünstige und niedrigschwellige Behandlungsmöglichkeiten einen deutlichen Schwerpunkt der Presse- und Öffentlichkeitsarbeit ausmachen sollten, da diese in der Medienberichterstattung kaum vorkommen. So könnte eine präventionsorientiertere und realitätsnähere Wahrnehmung von Gesundheitsthemen in der Bevölkerung und bei Entscheidungsträger*innen entstehen.

## Footnotes

1 Dies bedeutet, dass die Artikel Diabetes bzw. Depression nicht nur am Rande erwähnen oder als ein Beispiel anführen, sondern sich im Schwerpunkt mit diesen Erkrankungen auseinandersetzen. Die Bedingung für die Aufnahme in die Stichprobe aus ca. 29.000 Artikeln, die die Erkrankungen erwähnten, war, dass bei einer Zusammenfassung des Artikels in wenigen Worten definitiv die Worte Diabetes bzw. Depression enthalten sein mussten.
